# Supplementary material for: Bayesian refinement of protein structures and ensembles against SAXS data using molecular dynamics
Source: PLoS Comput Biol. 2017 Oct 18;13(10):e1005800. doi: 10.1371/journal.pcbi.1005800 (PMC5662244; doi:10.1371/journal.pcbi.1005800)
Supplement: S1 Archive — (BZ2) [file pcbi.1005800.s011.tar.bz2 › gromacs_4.62_bayesian_saxs_refi/share/html/online/genion.html]

genion


| genion Main Table of Contents |

**VERSION 4.6  
Sat 19 Jan 2013**

---

### Description

genion replaces solvent molecules by monoatomic ions at
the position of the first atoms with the most favorable electrostatic
potential or at random. The potential is calculated on all atoms, using
normal GROMACS particle-based methods (in contrast to other methods
based on solving the Poisson-Boltzmann equation).
The potential is recalculated after every ion insertion.
If specified in the run input file, a reaction field, shift function
or user function can be used. For the user function a table file
can be specified with the option -table.
The group of solvent molecules should be continuous and all molecules
should have the same number of atoms.
The user should add the ion molecules to the topology file or use
the -p option to automatically modify the topology.

The ion molecule type, residue and atom names in all force fields
are the capitalized element names without sign. This molecule name
should be given with -pname or -nname, and the
[molecules] section of your topology updated accordingly,
either by hand or with -p. Do not use an atom name instead!

Ions which can have multiple charge states get the multiplicity
added, without sign, for the uncommon states only.

With the option -pot the potential can be written as B-factors
in a .pdb file (for visualisation using e.g. Rasmol).
The unit of the potential is 1000 kJ/(mol e), the scaling be changed
with the -scale option.

For larger ions, e.g. sulfate we recommended using genbox.

### Files

| option | filename | type | description |
| --- | --- | --- | --- |
| **-s** | topol.tpr | Input | Run input file: tpr tpb tpa |
| **-table** | table.xvg | Input, Opt. | xvgr/xmgr file |
| **-n** | index.ndx | Input, Opt. | Index file |
| **-o** | out.gro | Output | Structure file: gro g96 pdb etc. |
| **-g** | genion.log | Output | Log file |
| **-pot** | pot.pdb | Output, Opt. | Protein data bank file |
| **-p** | topol.top | In/Out, Opt. | Topology file |

### Other options

| option | type | default | description |
| --- | --- | --- | --- |
| **-[no]h** | bool | no | Print help info and quit || **-[no]version** | bool | no | Print version info and quit || **-nice** | int | 19 | Set the nicelevel || **-xvg** | enum | xmgrace | xvg plot formatting: xmgrace, xmgr or none || **-np** | int | 0 | Number of positive ions || **-pname** | string | NA | Name of the positive ion || **-pq** | int | 1 | Charge of the positive ion || **-nn** | int | 0 | Number of negative ions || **-nname** | string | CL | Name of the negative ion || **-nq** | int | -1 | Charge of the negative ion || **-rmin** | real | 0.6 | Minimum distance between ions || **-[no]random** | bool | yes | Use random placement of ions instead of based on potential. The rmin option should still work || **-seed** | int | 1993 | Seed for random number generator || **-scale** | real | 0.001 | Scaling factor for the potential for -pot || **-conc** | real | 0 | Specify salt concentration (mol/liter). This will add sufficient ions to reach up to the specified concentration as computed from the volume of the cell in the input .tpr file. Overrides the -np and -nn options. || **-[no]neutral** | bool | no | This option will add enough ions to neutralize the system. In combination with the concentration option a neutral system at a given salt concentration will be generated. |

### Known problems

- Calculation of the potential is not reliable, therefore the -random option is now turned on by default.- If you specify a salt concentration existing ions are not taken into account. In effect you therefore specify the amount of salt to be added.

---

http://www.gromacs.org  
gromacs@gromacs.org
